# Recycled Aggregates Influence on the Mechanical Properties of Cement Lime-Based Mortars Part II

**DOI:** 10.3390/ma19071386

**Published:** 2026-03-31

**Authors:** Ioan Sorin Letiu, Catalin Saitis, Daniela Lucia Manea, Claudiu Aciu, Marioara Moldovan, Stanca Cuc, Ioan Petean

**Affiliations:** 1 Faculty of Civil Engineering, Technical University of Cluj-Napoca, 28 Memorandumului, 400114 Cluj-Napoca, Romania; ioan.letiu@campus.utcluj.ro (I.S.L.); daniela.manea@ccm.utcluj.ro (D.L.M.); claudiu.aciu@ccm.utcluj.ro (C.A.); 2 Department of Polymer Composites, Raluca Ripan Institute for Research in Chemistry, Babeș-Bolyai University, 30 Fantanele Street, 400294 Cluj-Napoca, Romania; stanca.boboia@ubbcluj.ro; 3 Faculty of Chemistry and Chemical Engineering, Babeș-Bolyai University, 11 Arany Janos Street, 400028 Cluj-Napoca, Romania; petean.ioan@gmail.com

**Keywords:** plastering mortars, sustainable development, old palster waste, microstructural analyses, SEM investigations

## Abstract

In the current framework of sustainable development, stricter international regulations are shaping waste management practices, particularly in the construction sector—one of the main generators of waste. This study investigates the reuse of deteriorated plaster waste from façades scheduled for rehabilitation, by partially replacing cement or aggregates in lime–cement mortars. Four mortar formulations were tested: a reference mix, one with 45% aggregate replacement by plaster waste, one with 10% cement replacement, and another combining the 45% aggregate replacement with polypropylene fibers. Both microstructural and macrostructural analyses were conducted to identify correlations between these levels when incorporating waste and fibers. Results show a decrease in compressive strength (36% for aggregate and 29% for cement replacement) and flexural strength (24% and 18%, respectively) as the replacement ratio increases. However, the inclusion of polypropylene fibers improved the mechanical performance. SEM analysis confirmed significant microstructural variations within the cementitious matrix due to waste incorporation. Despite reduced strength values, all mixtures remained within the limits defined by current standards, validating the feasibility of using plaster waste in mortars and emphasizing its potential to reduce construction waste and promote sustainable development.

## 1. Introduction

In the context of sustainable development, the construction sector plays a crucial role, significantly impacting the environment and natural resources. In the European Union, construction and demolition activities generate approximately 40% of the total waste produced, highlighting the importance of efficient waste management. Globally, it is estimated that the construction sector produces between 2 and 3 billion tons of waste annually, accounting for about 30% of total solid waste [1,2,3,4].

In this context, recycling and valorizing waste from construction and demolition (C&D) become strategic priorities. Recent studies have highlighted the use of recycled materials in the production of lime-cement-based mortars, replacing partially natural aggregates or cement with waste such as gypsum, concrete, or ceramics. These studies indicate that replacing aggregates with recycled waste can vary between 10% and 45%, depending on the type of material and the desired performance of the mortar [5,6,7].

The present study is an extension of the article “Recycled Aggregates Influence on the Mechanical Properties of Cement–Lime–Based Mortars” by Saitis Catalin et al. [8], with SEM analyses conducted at 7 and 14 days in addition to 28 days, allowing the microscopic evolution of the studied mortars over time to be tracked. While the earlier work included mechanical testing and mineralogical characterization through techniques such as X-ray diffraction (XRD) and chemical analysis, the current paper extends the investigation by providing a detailed SEM-based microstructural analysis. This complementary approach enables the direct observation of morphological features and microstructural mechanisms, offering a deeper understanding of the behaviour of mortars incorporating recycled plaster waste. The primary goal of this research is to perform a detailed microscopic investigation of the microstructural evolution of the cement-lime matrix at different curing ages and to directly correlate these changes with the mechanical behaviour of the investigated mortar samples. Although the reuse of construction and demolition waste has been widely studied, the microstructural mechanisms governing the incorporation of recycled plaster waste from façade renderings in lime–cement mortars remain insufficiently documented. Therefore, the present study aims to address this research gap by combining mechanical testing with SEM-based microstructural analysis in order to better understand the behaviour of such recycled mortar systems and to support the development of more sustainable construction materials.

Therefore, Table 1 provides a detailed overview of the state-of-the-art studies [8,9,10,11,12,13,14,15,16,17,18,19,20,21,22,23,24,25,26,27], emphasizing the replacement levels of conventional raw materials with recycled waste across various types of mortars. This synthesis is essential for understanding both the potential and the limitations of incorporating recycled materials into construction composites, supporting the development of more sustainable and resource-efficient building solutions.

- Microstructural observations

SEM analyses across the reviewed literature indicate that the integration of recycled materials—such as plaster waste, mortar powder, brick powder, or mixed demolition aggregates—affects the microstructure and interfacial transition zone (ITZ) of cement–lime mortars.

An increased porosity, irregular particle morphology, and weaker ITZ bonding were consistently observed when natural aggregates were partially replaced by recycled ones (Saitis Catalin et al. [8]; R. Ferreira et al. [11]; R. Samiei et al. [12]; A. Ruviaro et al. [13]; I. Raini et al. [20]; M. Stefanidou et al. [21]; V. Grigorjev et al. [22]). Conversely, the addition of fine recycled powders (plaster or rendering mortar waste) promotes matrix densification and pore refinement, as hydration products form around waste particles (A. Abadel et al. [10]; D. Koňáková et al. [18]; S. Zhang et al. [19]). Several studies, including those of A. Ruviaro et al. [13] and D. Koňáková et al. [18], confirm that over time, secondary hydration reactions lead to partial self-healing and improved internal cohesion.

At the micro-level, all research agrees that optimized replacement ratios (10–20%) enhance material compactness without critical degradation of the microstructure, while excessive waste content increases capillary connectivity and microcracking (R. Samiei et al. [12]; I. Raini et al. [20]).

- Macrostructural observations (mechanical behaviour)

At the macrostructural level, the inclusion of recycled aggregates and waste powders generally causes a moderate reduction in mechanical strength, particularly in compressive and flexural performance.

Reductions of 10–30% were reported depending on replacement level and waste type (S. Cătălin et al. [8,9]; A. Abadel et al. [10]; R. Ferreira et al. [11]; N. Garg et al. [14]; I. Raini et al. [20]; V. Grigorjev et al. [22]). Despite these decreases, most mixes remain within regulatory limits, making them suitable for non-structural and rendering applications (S. Cătălin et al. [8,9]; A. Abadel et al. [10]). Lightweight waste such as perlite reduces density while improving thermal insulation properties (M. Stefanidou et al. [21]).

Durability studies show that increased water absorption and permeability accompany higher waste content, yet long-term microstructural stabilization can occur through continued hydration (A. Ruviaro et al. [13]; N. Garg et al. [14]; D. Koňáková et al. [18]).

From a sustainability perspective, all reviewed works highlight the environmental and circular economy benefits of incorporating recycled waste, including reduced CO_2_ emissions and raw material conservation (R. Ferreira et al. [11]; N. Garg et al. [14]; M. Stefanidou et al. [21]; V. Grigorjev et al. [22]).

- Polypropylene fibers influence

The addition of polypropylene fibers to lime and cement–lime mortars significantly enhances ductility, toughness, and crack resistance (R. Illampas et al. [8]; A. Izaguirrea [9]; M. Pepe et al. [17]). At optimal fiber dosages (0.1–0.5% by volume), mortars display deflection-hardening behavior and improved residual flexural strength, while maintaining satisfactory workability (A. Izaguirrea [16]). SEM observations confirm effective fiber–matrix adhesion and microcrack bridging, which delays crack propagation (R. Illampas et al. [15]; M. Pepe et al. [17]). However, excessive fiber contents may lead to fiber clustering and local microvoids, compromising uniformity and reducing compressive strength (R. Illampas et al. [15]).

Overall, fiber reinforcement contributes to improved post-cracking performance and durability, particularly in lime-based matrices used for repair and strengthening applications (M. Pepe et al. [17]).

## 2. Materials and Methods

In the experimental program of the present study, all materials were selected and processed in full compliance with current regulatory standards [28,29,30,31]. By exploring the reuse of plaster mortar waste as a functional component in new mortar formulations, this research promotes sustainable construction practices that reduce the environmental burden associated with demolition activities, while supporting circular economy principles in the building industry.

The plaster waste employed originated from the restoration of an old facade located in Cluj-Napoca, Romania. During the renovation process, the degraded exterior plaster was completely removed from the underlying brick masonry. The collected waste was carefully extracted, separated from other demolition residues, and sorted into predefined particle size fractions to enable its effective use as a partial replacement for natural aggregates or cement in plaster mortars.

The sorting, preparation, and curing of the mortar specimens until reaching the designated testing intervals of 7, 14, and 28 days, were carried out in the Building Materials Laboratory of the Faculty of Civil Engineering, Technical University of Cluj-Napoca. Meanwhile, the mechanical tests and SEM analyses of the specimens were conducted at the Raluca Ripan Institute for Research in Chemistry, Babeș-Bolyai University, Cluj-Napoca.

### 2.1. Sample Preparation

In current research, 4 types of recipes have been studied as shown in Table 2: CSIII R.S. (reference recipe without plaster waste), CSIII 10% cement (10% of the cement was replaced with plaster waste), CSIII 45% aggregates (45% of the natural aggregates were replaced with plaster waste), and CSIII 45% aggregates + pp fibers.

According to Table 2, the mortar specimens were produced using standardized materials, including Portland cement type CEM II/A-S 52.5 R (Holcim Extradur, Holcim, Câmpulung, Romania), stored in accordance with SR EN 196 [31] requirements, hydrated lime (Carmeuse Supercalco M, Carmeuse, Romania), and natural river sand classified into three granular fractions: 0–0.5 mm, 0.5–1.0 mm, and 1.0–2.0 mm. Plastering mortar waste collected from the façade of an old building in Cluj-Napoca, Romania, was similarly sorted into identical particle size ranges, exhibiting a loose bulk density of 1066.20 kg/m^3^. Potable water was used for mixing, with dosage adjusted to achieve the desired workability for each mortar formulation.

The typical chemical composition was determined by RIR method and it is presented in Table 3.

Aggregate minerals mainly consist of quartz and calcite, with minor traces of muscovite associated with the granular component of the old plaster mortar. The lime—Portland cement binder develops cohesion through hydration products that bind the aggregate particles, contributing to the mechanical strength of the mortar.

### 2.2. Investigation Methods

All mortar compositions investigated in this study, including both the reference mix and those incorporating plaster waste, were prepared and tested in accordance with the relevant European standards. Aggregates were evenly divided into particle size fractions of 0–0.5 mm, 0.5–1.0 mm, and 1.0–2.0 mm, while the quantities of cement, lime, aggregates, and water were accurately measured prior to mixing. The materials were blended using an automatic mortar mixer to ensure complete homogenization, after which the fresh mortar was subjected to consistency testing according to SR EN 1015-3 [32] and apparent density determination according to SR EN 1015-6 [33]. The mixtures were then cast into standardized prismatic molds (40 × 40 × 160 mm), compacted on a vibrating table, and cured under controlled conditions at 20 ± 2 °C and 65–70% relative humidity, in compliance with SR EN 196 [31]. In addition to mechanical strength testing at 7, 14, and 28 days, microscopic investigations using Scanning Electron Microscopy (SEM) were conducted at the same curing intervals to examine the microstructural evolution of the cementitious matrix as shown in Table 4.

**Figure 1:**
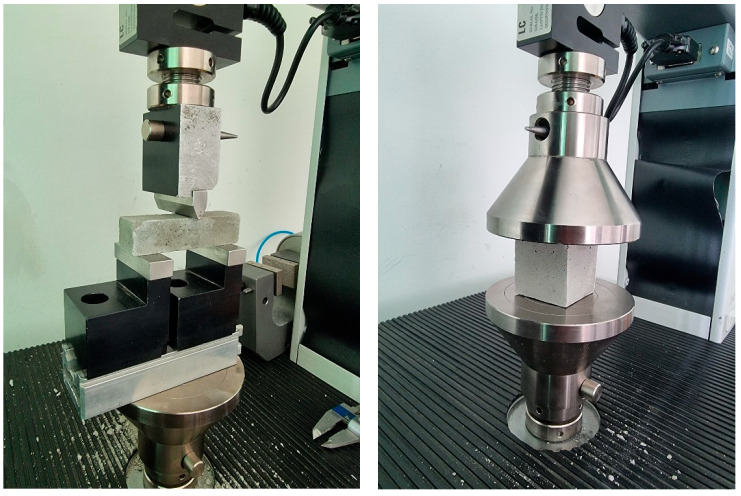
Bending and compressive strength determinations.

As presented in Table 4, the mortars were characterized in both the fresh and hardened states with respect to key physical, mechanical (Figure 1), and microstructural properties, using the specialized equipment.

The apparent density was determined in the fresh state using a cylindrical vessel with a volume of 1 L, following the procedure described in SR EN 1015-6 [33]. The container was filled with freshly mixed mortar, properly compacted, and the excess material was removed. The apparent density was calculated as the ratio between the net mass of the mortar and the vessel volume. In the hardened state, the apparent density was determined for three specimens at each curing age (7, 14, and 28 days) by weighing the samples and dividing their mass by the actual prism volume according to SR EN 1015-10 [34].

The consistency of the fresh mortar was evaluated in accordance with SR EN 1015-3 [32], using a flow table test. The mortar was placed on a metal plate using a truncated cone mold, which was then carefully lifted to allow the sample to retain its shape. The table was dropped from a controlled height, causing the mortar to spread. The final consistency value was obtained as the arithmetic mean of two perpendicular diameters measured across the spread sample. The water–cement ratio was adjusted based on the fluidity of each sample, considering that the incorporation of old plaster waste requires a higher water content to achieve appropriate consistency.

The flexural (bending) strength was determined according to SR EN 1015-11 [35], on three prismatic specimens for each curing age using a standard three-point bending setup. The strength was calculated using the formula specified in the standard, where the breaking force is multiplied by 1.5 times the span length between the supports and divided by the cube of the prism side length.

The compressive strength was determined on the resulting halves of the specimens tested in bending, using a compression testing machine in compliance with SR EN 1015-11 [35]. The value was calculated as the ratio between the maximum applied load at failure and the cross-sectional area of the prism.

The adhesion to the substrate was evaluated using the pull-off method in accordance with SR EN 1015-12 [36]. Circular traction discs were bonded to the mortar surface using an epoxy adhesive, and the tensile load was gradually applied until detachment occurred. The recorded value represented the adhesion strength to the support layer.

Scanning Electron Microscopy (SEM) investigations were performed using an Inspect S microscope (FEI Company, Hillsboro, OR, USA), operated in low-vacuum mode at an accelerating voltage of 25 kV. The microstructure and fractography of the mortars were examined at magnifications of 50×, 100×, 500×, 1000×, 2000×, and 5000×. These analyses allowed the observation of microstructural features such as pore morphology, particle distribution, and the interfacial transition zone between the binder matrix and recycled plaster aggregates.

All experimental results were analyzed using a one-way ANOVA with a significance level of 0.05. For cases in which statistically significant differences were identified, Tukey’s post-hoc test was employed to determine pairwise group differences.

## 3. Results and Discussion

### 3.1. Fresh State Characteristics Results

Fresh state results obtained for apparent density and consistency are shown in Table 5 as it follows:

The apparent density results for both cases—where aggregates and cement were partially substituted with plaster waste—showed consistent values around 2000 kg/m^3^, in accordance with the specifications outlined in the relevant standards SR EN 1015-3 [32].

The consistency of the mortars, remained constant between 15–20 cm, which is within the range prescribed by the applicable technical standards SR EN 1015-6 [33]. For the mixture containing 1.5% polypropylene fibers, the same amount of water was used as in the formulation where only 45% of the aggregates were replaced. However, due to the presence of fibers, the consistency value decreased by approximately 14%, confirming the influence of fiber addition on the workability of the fresh mortar.

Mechanical and microstructural analyses show that incorporating old plastering waste generally reduces the macroscopic and microscopic performance of the mortars, consistent with literature reports indicating that higher replacement levels of cement or aggregates with waste lead to diminished material properties.

**Figure 2:**
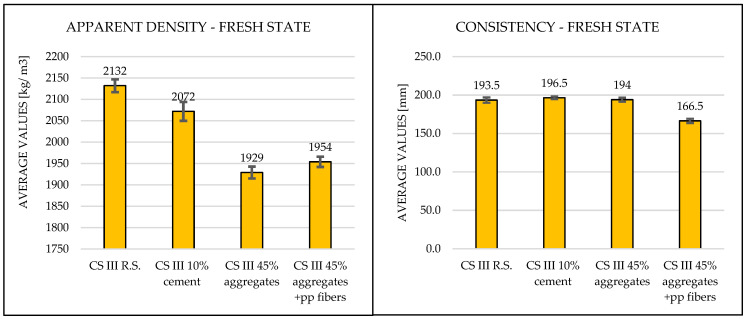
Apparent density and consistency graphical results in fresh state.

Figure 2 illustrates the fresh state properties of the analyzed mortars.

The fresh apparent density of the reference CS III R.S. mortar was 2132 kg/m^3^, within the typical 2100–2200 kg/m^3^ range for cement–lime mortars (Saitis et al. [8]; Ferreira et al. [11]). Replacing 10% of cement with plaster waste (CS III 10% cement) slightly reduced density to 2072 kg/m^3^, consistent with minor reductions reported by Abadel et al. [10] and Saitis et al. [9]. Substituting 45% of aggregates (CS III 45% aggregates) lowered density further to 1929 kg/m^3^ due to the higher porosity and lower specific gravity of recycled particles, in line with Saitis et al. [8], Ruviaro et al. [13], and Ferreira et al. [11]. Adding 1.5% polypropylene fibers (CS III 45% aggregates + PP fibers) slightly increased density to 1954 kg/m^3^, reflecting partial densification as observed by Illampas et al. [15] and Izaguirrea et al. [16].

Consistency of the reference mix was 193.5 mm, within the 150–200 mm typical range for plastering mortars (Saitis et al. [9]; Abadel et al. [10]). The 10% cement replacement showed negligible change (196.5 mm), while 45% aggregate substitution slightly reduced flow to 194 mm, consistent with higher water absorption of recycled aggregates (Saitis et al. [8]; Ferreira et al. [11]). Incorporating fibers decreased consistency to 166.5 mm, about 14% lower than the fiber-free mix, reflecting the water-absorbing effect and flow restriction caused by polypropylene fibers, as noted by Illampas et al. [15] and Izaguirrea et al. [16].

### 3.2. Hardened State Characteristics Results

The determination of physical and mechanical properties like apparent density, bending and compressive strength, and adhesion to the substrate for the mortars was carried out at 7, 14, and 28 days after casting. The results are presented in Table 6, covering both cases—the replacement (%) of aggregates and the replacement (%) of cement with plaster waste.

Regarding apparent density, the results show a gradual decrease from one testing age to the next, primarily due to water loss through evaporation. The reduction in density observed in mortars containing plaster waste confirms that the cement hydration process proceeds effectively, and the hardening of the mortar is not significantly affected by the inclusion of waste.

For flexural (bending) strength, the results indicate a consistent trend of decreasing values as the percentage of waste increases. The mortars demonstrate a greater capacity to accommodate higher replacement levels of aggregates compared to cement. Moreover, the addition of polypropylene fibers improves the 28-day flexural strength by approximately 11% compared to the mix without fibers.

In terms of compressive strength, a similar trend of decreasing values with increasing waste content is observed. The inclusion of polypropylene fibers enhances the 28-day compressive strength by approximately 14% relative to the reference mix without fibers.

The results for adhesion to the substrate indicate some negative influence from the inclusion of old plaster waste; however, the values remain within acceptable limits and comply with the requirements of the relevant standards.

**Figure 3:**
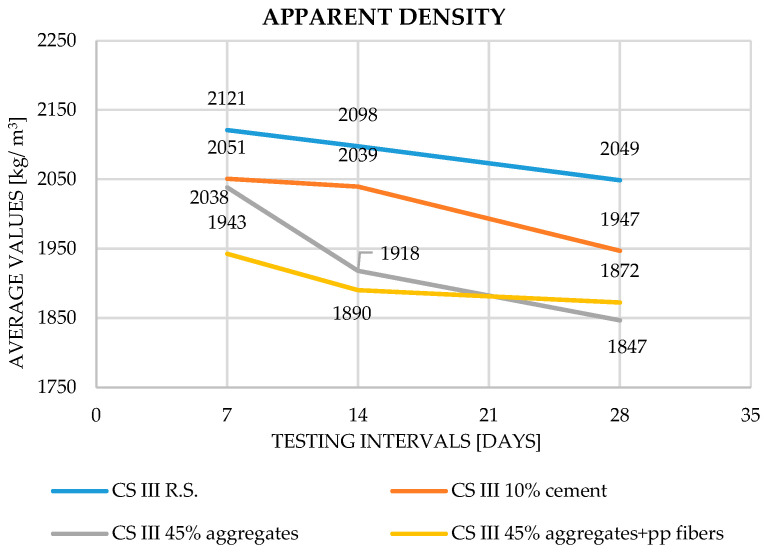
Apparent density—evolution of results during the curing process.

**Figure 4:**
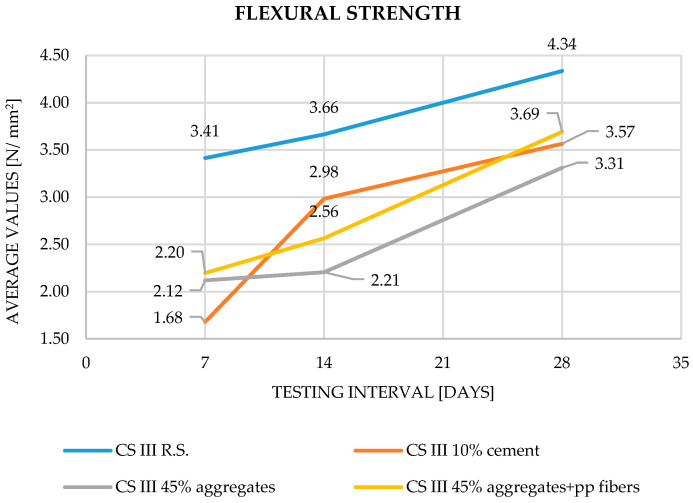
Flexural Strength—evolution of results during the curing process.

**Figure 5:**
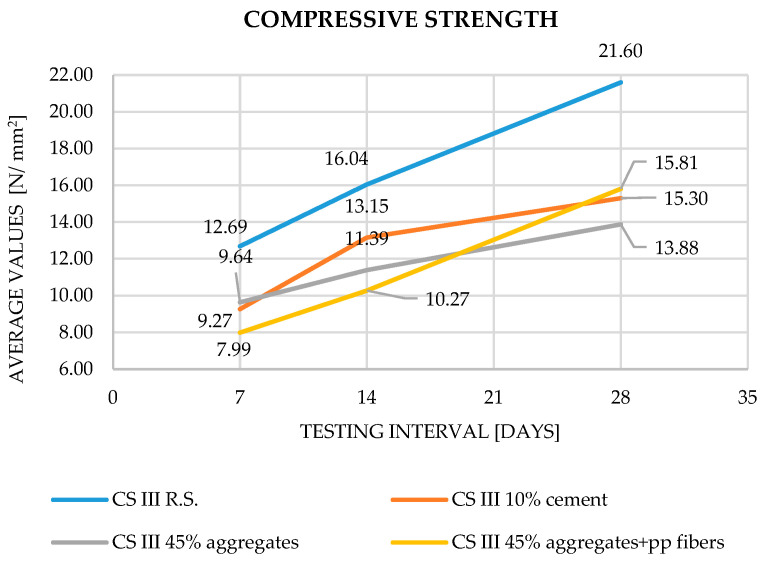
Compressive strength—evolution of results during the curing process.

**Figure 6:**
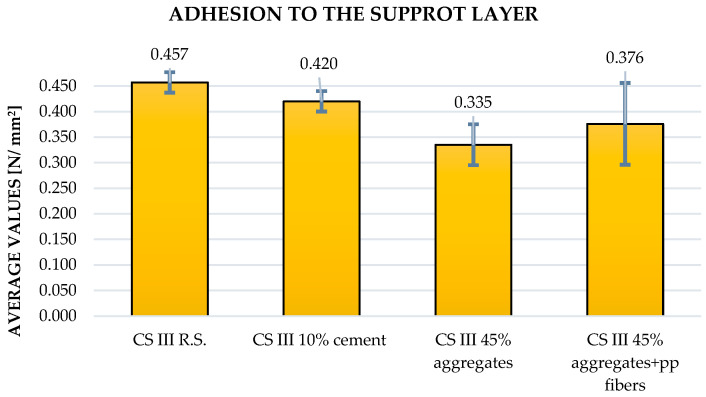
Adhesion to the support layer—28 days results.

Across all parameters, the experimental results are in close agreement with trends reported in the literature. Increasing substitution levels of cement or aggregates with plaster waste consistently resulted in reduced densities and mechanical performance (Saitis et al. [8,9]; Abadel et al. [10]; Ruviaro et al. [13]; Ferreira et al. [11]). However, the inclusion of polypropylene fibers counteracted these reductions, improving both tensile and compressive strengths by 10–15%, in full accordance with Illampas et al. [15] and Izaguirrea et al. [16]. The obtained results for apparent density, flexural strength, compressive strength and adhesion to the support layer can be observed in Figure 3, Figure 4, Figure 5 and Figure 6.

In terms of apparent density, all mixes showed a gradual decrease in density over curing time, attributed to water evaporation.

The reference mortar decreased from 2210 kg/m^3^ (7 days) to 2044 kg/m^3^ (28 days), in line with Saitis et al. [8] and Ferreira et al. [11]. For the 10% cement replacement, density values ranged from 2051 to 1947 kg/m^3^, confirming the trend noted by Abadel et al. [10], who reported similar reductions due to lower particle density and increased micro-porosity. In the 45% aggregate replacement mix, density dropped more significantly (2038 → 1847 kg/m^3^), consistent with Saitis et al. [8], Ruviaro et al. [13], and Grigorjev et al. [22], all of whom observed lower densities when large portions of aggregates were replaced with recycled materials. When PP fibers were incorporated, density values at 28 days (1872 kg/m^3^) indicated slightly improved compactness, a behavior also reported by Illampas et al. [15], suggesting that fibers limit microcrack formation during drying.

In case of flexural strength, the reference mortar reached 4.34 N/mm^2^ at 28 days, consistent with typical values of 3–5 N/mm^2^ for cement–lime mortars (Saitis et al. [8]; Ferreira et al. [11]). For the 10% cement replacement, the 28-day flexural strength was 3.57 N/mm^2^, slightly below the reference, confirming the observations of Abadel et al. [10], who reported modest reductions when rendering waste replaced part of the cement. The 45% aggregate replacement yielded 3.31 N/mm^2^, about 25–30% lower than the control, consistent with findings by Saitis et al. [8], Ruviaro et al. [13], and Garg and Shrivastava [14], who associated this behavior with weaker interfacial bonding between the binder matrix and recycled aggregates. Adding PP fibers increased flexural strength to 3.69 N/mm^2^, reflecting an ≈11% improvement relative to the unreinforced mix. Similar enhancements were observed by Illampas et al. [15] and Izaguirrea et al. [16], who linked fiber addition to crack-bridging and stress redistribution effects.

Compressive strength results showed that the reference mortar achieved 21.6 N/mm^2^ at 28 days, consistent with standard cement–lime mortar ranges (Saitis et al. [8]; Ferreira et al. [11]). For the 10% cement replacement, the compressive strength decreased to 15.3 N/mm^2^, representing a reduction of approximately 25%, in agreement with Abadel et al. [10], who observed similar effects due to partial substitution of the hydraulic binder. The 45% aggregate replacement mortar recorded 13.88 N/mm^2^, a 35–40% decrease, corroborating the results of Saitis et al. [8], Ruviaro et al. [13], and Grigorjev et al. [22], where recycled aggregates led to diminished load-bearing capacity. When PP fibers were added, the compressive strength increased to 15.81 N/mm^2^, an improvement of roughly 14%, confirming the reinforcing contribution highlighted in Illampas et al. [15] and Izaguirrea et al. [16].

Adhesion to the substrate was tested at the age of 28 days after the mortars preparation and the reference mortar exhibited an adhesion strength of 0.457 N/mm^2^, falling within the normative limits (0.3–0.6 N/mm^2^) specified in specialized standards and consistent with Saitis et al. [8] and Ferreira et al. [11]. The 10% cement replacement sample recorded 0.420 N/mm^2^, comparable to the results of Abadel et al. [10], indicating limited influence of small binder substitutions. The 45% aggregate replacement sample decreased to 0.335 N/mm^2^, similar to the values obtained by Saitis et al. [8] and Ruviaro et al. [13], due to increased porosity and weaker bonding at the interface. The incorporation of PP fibers slightly improved adhesion to 0.376 N/mm^2^, confirming the positive interfacial effects observed in Illampas et al. [15] and Izaguirrea et al. [16], where fibers improved mechanical interlock and crack control.

Overall, these mechanical findings support the conclusions of Purchase [5] and Sakthibala et al. [6], confirming that controlled incorporation of plaster waste—especially when coupled with fiber reinforcement—can contribute to sustainable, circular-economy-based mortar formulations without critically compromising mechanical integrity.

The microscopic investigations performed through Scanning Electron Microscopy (SEM) provided valuable insights into the internal structure and morphology of the analyzed mortars. By observing the microstructural evolution at different curing ages, the influence of recycled plaster waste and polypropylene fibers on the compactness and bonding quality within the matrix could be identified and compared with findings reported in the literature. The identification of hydration products such as portlandite, ettringite, and tobermorite was based primarily on their characteristic morphologies observed in the SEM images and on their typical occurrence in hydrated Portland cement systems. In this context, the term tobermorite is used to describe lamellar C–S–H structures exhibiting a morphology commonly associated with tobermorite-type phases formed during cement hydration.

**Figure 7:**
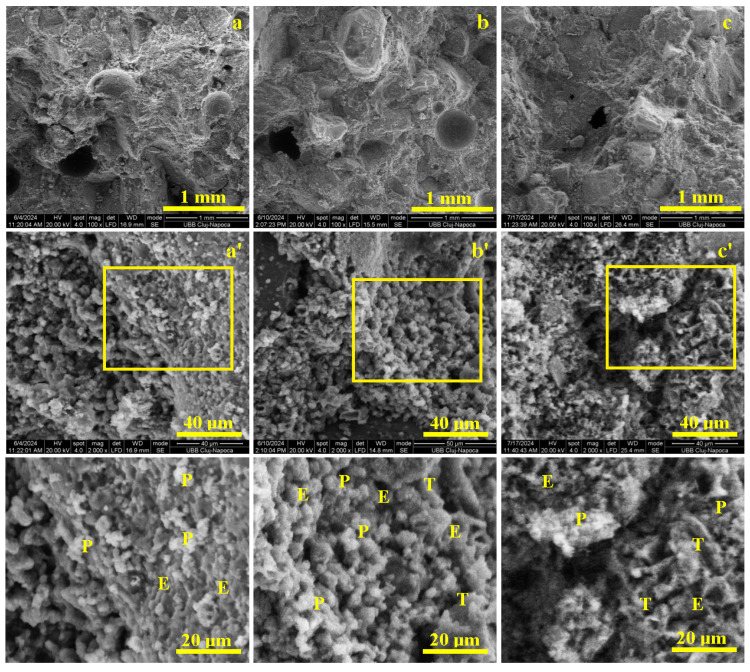
SEM results of the macroscopic appearance of the CS III R.S., mortar samples cured for: (**a**) 7 days, (**b**) 14 days, and (**c**) 28 days. (**a’**) is Microstructural details of 7 Days, (**b’**) is Microstructural details of 14 Days, (**c’**) is Microstructural details of 28 Days. Microstructural details are marked with ’, and the hydrated compounds are indicated in the highlighted zones as follows: T—Tobermorite, P—Portlandite, E—Ettringite.

The CS III R.S. sample consists of the standard cement–aggregate mixture without any plaster waste additions. Consequently, mixing with the standard amount of water converts the cement powder into a workable paste that uniformly coats and binds the quartz sand particles, resulting in a compact and homogeneous composite as shown in Figure 7.

After 7 days of curing, the early-stage hardening of the mortar is clearly observed in Figure 7a. The cementitious matrix surrounds the quartz aggregates, but the aggregate–matrix bond remains weak due to the predominance of fine portlandite nodules (1–5 µm), visible in Figure 7a’, together with acicular ettringite crystals. Although these hydration products initiate matrix cohesion, under flexural and compressive loading the portlandite-rich interfaces fail easily, leading to aggregate delamination and low early mechanical performance, as indicated by the smooth fracture surfaces.

After 14 days of curing, the aggregates become more strongly embedded within the cementitious matrix, as shown in Figure 7b. A continuous and compact layer of hydration products coats the aggregate surfaces, corresponding to the maturation of portlandite and ettringite and the onset of bisphenoidal tobermorite formation. The microstructural detail in Figure 7b’ highlights tabular tobermorite crystals (≈5–15 µm), which enhance interfacial bonding and matrix cohesion. As a result, stress transfer improves, the aggregates remain intact, and both flexural and compressive strengths increase, with fracture surfaces exhibiting irregular, chipped areas rather than clean debonding.

After 28 days of curing, the sample shows a highly compact and mature microstructure (Figure 7c). The cementitious matrix fully anchors the quartz aggregates through a dense and continuous network of well-developed tobermorite crystals, as illustrated in Figure 7c’. At this stage, fracture propagation occurs predominantly through the cement matrix instead of along the aggregate–matrix interface, confirming the attainment of structural maturity and peak mechanical performance.

Thus, the high mechanical performance of the standard mix is attributed to the quality of the quartz sand aggregate and the well-hydrated Portland cement paste, which promotes the sequential formation of hydration products—portlandite, ettringite, and tobermorite. Among these, tobermorite (a calcium silicate hydrate phase) provides the optimal cohesion and mechanical integrity of the mortar structure.

In conclusion, SEM analysis of the CS III R.S. sample highlights a clear hydration progression from portlandite-dominated early stages to mature tobermorite formation. At 7 days, fine portlandite nodules (1–5 µm) and initial acicular ettringite result in weak aggregate–matrix bonding, reflected by smooth detachment surfaces. By 14 days, the development of tabular and bisphenoidal tobermorite crystals (5–15 µm) significantly improves cohesion and load transfer, consistent with observations reported by Saitis et al. [8] and Ferreira et al. [11]. At 28 days, a dense C–S–H/tobermorite-dominated matrix is formed, in line with findings by Koňáková et al. [18] and Zhang et al. [19], leading to matrix-controlled fracture propagation and explaining the high mechanical performance of the reference mix.

**Figure 8:**
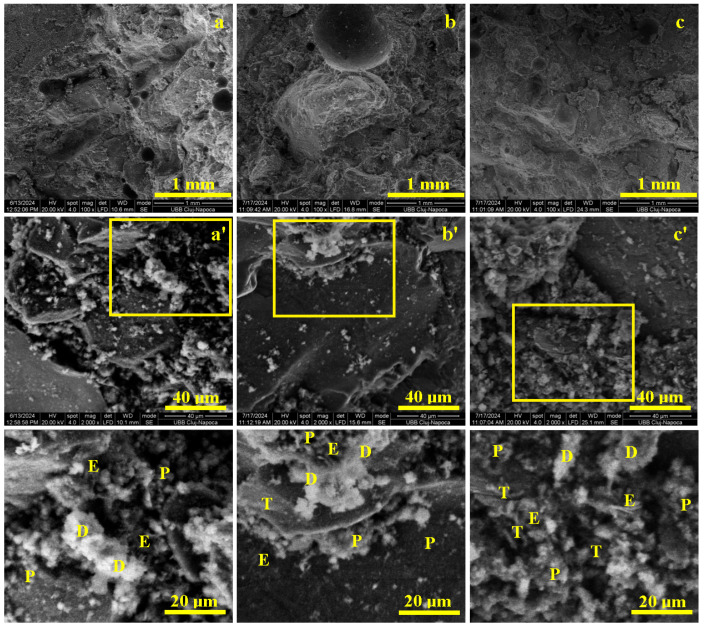
SEM results of the macroscopic appearance of the CS III 10% cement, mortar samples cured for: (**a**) 7 days, (**b**) 14 days, and (**c**) 28 days. (**a’**) is Microstructural details of 7 Days, (**b’**) is Microstructural details of 14 Days, (**c’**) is Microstructural details of 28 Days. Microstructural details are marked with ’, and the hydrated compounds are indicated in the highlighted zones as follows: T—Tobermorite, P—Portlandite, E—Ettringite, and D—plaster waste.

In case of CS III 10% cement sample, at microstructural level as shown in Figure 8, the plaster waste particles mix intimately with the cement grains and simultaneously interact with water. This process requires an additional 20 L of water compared to the standard mix to properly wet the highly dusty plaster waste and achieve similar consistency. The wet incorporation of the plaster powder into the cement paste is essential to ensure microstructural homogenization.

Grinding the plaster waste induces microcracking of the old cement layer covering the recycled particles, facilitating water penetration and in-situ hydration during Portland cement curing. SEM observations on fractured specimens (Figure 8) show that the finely milled plaster waste is homogeneously dispersed within the cement paste, which uniformly binds the quartz sand aggregates, acting as a continuous matrix.

After 7 days, the matrix is solidified and the aggregates are immobilized (Figure 8a), but the immature bonding leads to partial aggregate delamination under load, evidenced by smooth, spherical detachment surfaces and explaining the low early strengths. By 14 days, a denser structure develops (Figure 8b), with aggregates more firmly embedded and coated by hydration products, as indicated by chipping marks and intact cement layers, enabling improved load transfer and higher mechanical performance. At 28 days, the matrix reaches full maturity (Figure 8c), and failure occurs through matrix tearing rather than aggregate debonding, resulting in significantly increased flexural and compressive strengths.

Microstructurally, Figure 8a’ reveals fractured plaster waste clusters (20–60 µm) penetrated by cement paste, along with fine portlandite particles (1–5 µm) and early acicular ettringite, which provide initial cohesion after 7 days. At 14 days, bisphenoidal tobermorite lamellae (3–10 µm) develop more extensively (Figure 8b’), reinforcing the portlandite bridges and strongly consolidating the aggregate–matrix interface. By 28 days (Figure 8c’), a dense network of tobermorite, intergrown with ettringite and portlandite, ensures complete microstructural maturity.

Overall, replacing 10% of cement with finely ground plaster waste slightly delays the formation of advanced hydration products but does not hinder long-term performance. Once maturity is reached, the microstructure supports good mechanical strength, confirming the suitability of this recycled material for practical applications.

In the CS III 10% cement mix, where 10% of cement was replaced with finely ground plaster waste, SEM analysis shows that higher water demand was needed to achieve comparable consistency. At 7 days, the microstructure is dominated by early portlandite formation and incipient ettringite, leading to partial aggregate delamination under load. By 14 days, the development of 3–10 µm tobermorite plates strengthens the aggregate–matrix interface, while at 28 days the matrix reaches full maturity, with a dense intergrowth of tobermorite, portlandite, and ettringite, resulting in mechanical performance comparable to the reference mix. These observations are consistent with findings by Abadel et al. [10], Koňáková et al. [18], and Ruviaro et al. [13], who report delayed early hydration but progressive matrix densification and adequate final mechanical properties when fine waste powders partially replace cement.

The CS III 45% aggregates sample involves replacing 45% of the natural quartz sand with crushed and finely sieved plaster waste. The primary goal of this substitution is to assess the morphological and microstructural compatibility between plaster waste particles and the hydrated cement–lime binder.

**Figure 9:**
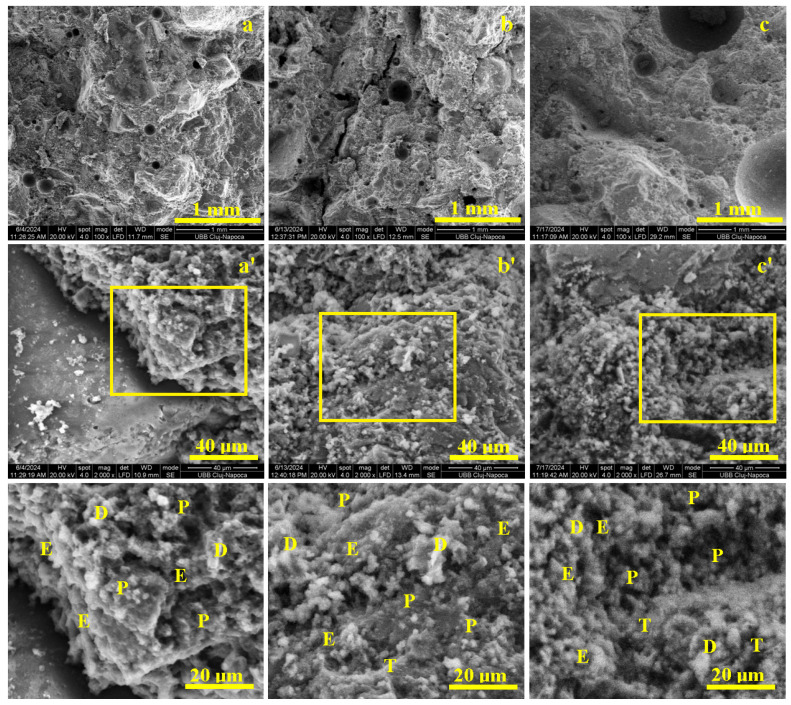
SEM results of the macroscopic appearance of the CS III 45% aggregate, mortar samples cured for: (**a**) 7 days, (**b**) 14 days, and (**c**) 28 days. (**a’**) is Microstructural details of 7 Days, (**b’**) is Microstructural details of 14 Days, (**c’**) is Microstructural details of 28 Days. Microstructural details are marked with ’, and the hydrated compounds are indicated in the highlighted zones as follows: T—Tobermorite, P—Portlandite, E—Ettringite, and D—plaster waste.

As seen in Figure 9, at the microstructural level, the plaster waste particles act as filler and partial aggregate substitute, forming direct contact surfaces with the cementitious paste. Due to their porous and irregular nature, the waste particles tend to absorb water during mixing, which slightly increases the overall water demand of the mixture to maintain the required workability. The fresh state density of 1929 kg/m^3^ and consistency of 194 mm reflect the lighter and more porous character of the recycled aggregate, compared to the reference mix.

The dust from the plaster waste partially mixes with the cement paste, forming localized blends, but the high content of recycled aggregate also creates dry interactions, resulting in microstructural imperfections (Figure 9a). These appear as dry fronts where the cement paste fails to fully coat the recycled aggregates, visible as whitish edges. Figure 9a’ shows a particle completely lacking a new cement layer, though remnants of the original hardened paste remain, while other areas display good coverage with portlandite and fine ettringite embedding the dust particles into a compact mass.

After 7 days, these dry zones offer minimal resistance under load, destabilizing the fresh cement layer and explaining the low mechanical performance (tensile = 2.12 N/mm^2^; compressive = 9.64 N/mm^2^). By 14 days, well-cemented areas consolidate further with tobermorite sheets (Figure 9b), enhancing bonding between moistened aggregates and the matrix. Mixed domains of ettringite and tobermorite interlock local voids (Figure 9b’), improving mechanical strength compared to 7 days (tensile = 2.21 N/mm^2^; compressive = 11.39 N/mm^2^), though dry zones still act as weak points.

At 28 days, the matrix reaches microstructural maturity (Figure 9c). Initially dry regions trigger fracture, tearing the cementitious matrix and pulling out some quartz particles, forming pitted cavities. Figure 9c’ shows lamellar tobermorite (8–10 µm × 1–1.5 µm) tightly intergrown with ettringite and portlandite, correlating with significant strength gains (tensile = 3.31 N/mm^2^; compressive = 13.88 N/mm^2^; adhesion = 0.335 N/mm^2^).

Overall, the high plaster waste content increases interfacial transition zones and microvoids, creating preferential crack paths. Nevertheless, well-distributed hydration products ensure sufficient load transfer, confirming that plaster waste can effectively replace up to 45% of aggregate when water and curing are properly managed.

In conclusion, in the CS III 45% aggregates sample, where 45% of quartz was replaced with plaster waste, SEM images show heterogeneous interfaces with “dry” fronts at 7 days, where cement failed to fully coat recycled aggregates, leaving microvoids and irregular fracture surfaces. By 14 days, well-cemented areas coexist with weak zones, partially reinforced by tobermorite and ettringite. At 28 days, further hydration densifies the matrix, but initial weak spots persist, slightly reducing cohesion compared to the reference mix. These findings align with Saitis et al. [8], Ruviaro et al. [13], Ferreira et al. [11], Samiei et al. [12], and Raini et al. [20], confirming that high recycled aggregate content increases ITZ volume and porosity, causing early microstructural weaknesses that improve over time but are not fully eliminated.

**Figure 10:**
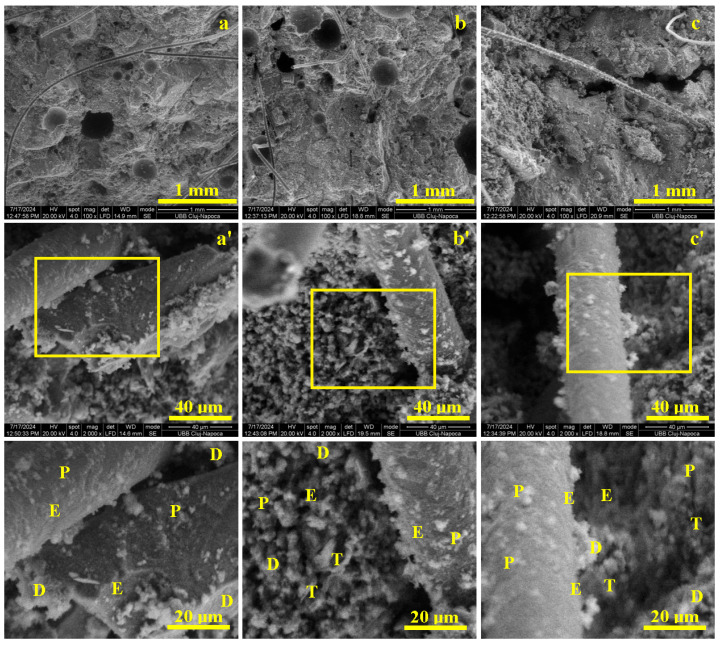
SEM results of the macroscopic appearance of the CS III 45% aggregate + pp fibers, mortar samples cured for: (**a**) 7 days, (**b**) 14 days, and (**c**) 28 days. (**a’**) is Microstructural details of 7 Days, (**b’**) is Microstructural details of 14 Days, (**c’**) is Microstructural details of 28 Days. Microstructural details are marked with ’, and the hydrated compounds are indicated in the highlighted zones as follows: T—Tobermorite, P—Portlandite, E—Ettringite, and D—plaster waste.

For CS III 45% aggregates + pp fibers, the addition of fibers provides the advantage of creating microstructural bridging throughout the recycled aggregate. Figure 10a shows a fiber positioned horizontally along the fracture plane of the sample cured for 7 days. Its length exceeds the field of view, and its diameter is approximately 40 µm. Its slightly sinuous configuration, as shown in Figure 10 contributes to improved cohesion within the mortar matrix. In the lower-left corner of Figure 10a, the broken ends of fibers that failed under mechanical loading can be observed.

Flexural loading induces axial tension in the lower part of the specimen, placing fibers there under tensile stress, while the upper region is comparatively compressed. Under compression, expansive stress along the weakest mid-section induces axial stress in the fibers. In the fresh state, the mortar shows reduced flow (166.5 mm, ~14% lower than the mix without fibers) and apparent density of 1954 kg/m^3^, due to the high specific surface area of polypropylene fibers absorbing water and restricting mobility, though this enhances structural cohesion upon hardening.

After 7 days, fractures occur mainly through dry-front zones where cement did not fully wet the recycled aggregates. Fibers provide microstructural bridging, delaying failure. Figure 10a’ shows delamination of fiber surfaces from partially consolidated cement, facilitating pull-out rather than rupture. Fibers adhere via portlandite and acicular ettringite, resulting in moderate early strengths (tensile = 2.20 N/mm^2^; compressive = 7.99 N/mm^2^).

By 14 days, fibers are more firmly embedded (Figure 10b), absorbing tensile loads and rupturing under stress, while tobermorite formation improves fiber–matrix bonding (Figure 10b’). Fiber necking reduces diameters to ~30 µm at rupture, correlating with increased tensile (2.56 N/mm^2^) and compressive strength (10.27 N/mm^2^).

At 28 days, the cementitious matrix reaches maturity, and while initially dry zones remain weak points, fibers bridge cracks (~40 µm wide, >2 mm long, Figure 10c), maintaining cohesion. Failure occurs mainly through matrix rupture rather than fiber–matrix debonding. Overall, the fiber-reinforced CS III 45% plaster waste samples exhibit improved mechanical performance at all ages, with 28-day tensile, compressive strength, and adhesion increasing to 3.69 N/mm^2^, 15.81 N/mm^2^, and 0.376 N/mm^2^, respectively—representing ~11% and 14% gains over the corresponding mix without fibers.

In conclusion, the CS III 45% aggregates + pp fibers sample demonstrates the influence of fibers on microstructural behavior. These results are in real agreement with Illampas et al. [15], Izaguirrea et al. [16], and Pepe et al. [17], who show that polypropylene fibers act as crack-bridging elements and are progressively reinforced by hydration products, thereby improving post-crack behavior. Additionally, the observations of fiber necking and rupture are fully consistent with the mechanisms described in the literature, highlighting that fibers effectively enhance load redistribution and energy dissipation in mortars containing high percentages of recycled aggregates.

## 4. Conclusions

SEM results agree well with literature trends for hydration, ITZ development, and the effects of recycled aggregates and fibers. The reference mix shows the typical portlandite–ettringite–tobermorite sequence and uniform densification, while 10% cement replacement slightly delays early hydration but achieves good later-age cohesion. High aggregate replacement (45%) leads to heterogeneous zones that are partly healed with time, and polypropylene fibers provide effective crack-bridging and load redistribution. Overall, the study confirms published findings and documents microstructural evolution at 7, 14, and 28 days.

In summary, considering all macroscopic and microscopic investigations conducted on the mortar samples, the following conclusions can be drawn:

1. Fresh state properties (consistency and apparent density)—Increasing plaster waste content leads to reduced consistency and fresh apparent density due to the higher porosity and lower specific gravity of recycled particles, in agreement with previous studies.
2. Compressive strength—decreases with plaster waste addition because recycled particles generate micro-defects and weak ITZs, which act as preferential failure paths. Fibre reinforcement partially compensates for this effect by bridging cracks and delaying their propagation.
3. Flexural strength—particularly sensitive to weak plaster–cement interfaces. SEM shows crack initiation along these boundaries, while fibres enhance flexural performance by bridging cracks through pull-out at early ages and rupture at later ages.
4. Adhesion to the substrate—Adhesion decreases with increasing plaster waste content but remains within normative limits, indicating that recycled plaster does not critically compromise bond performance.
5. Apparent density—Higher plaster waste replacement reduces hardened density due to increased dry fronts and uncoated recycled particles, as confirmed by SEM. This reduced compactness correlates with lower mechanical strength.
6. SEM analyses—at early ages, incomplete wetting of recycled particles produces dry fronts and weak ITZs that increase porosity and reduce strength. With ongoing hydration, the formation of tobermorite, ettringite, and portlandite consolidates the matrix and improves cohesion. In fibre-reinforced mixes, polypropylene fibres bridge cracks and delay their propagation, linking the observed microstructural evolution to the improved mechanical performance
7. Integrated interpretation of mechanical and SEM results—mechanical and SEM results show that increasing plaster waste reduces mechanical performance mainly due to weaker ITZs. SEM evidence indicates that fibres partially compensate for these effects by improving microstructural continuity and delaying crack propagation. Lower strengths correspond to poorly bonded recycled particles, while higher strengths are associated with denser C–S–H formation and better fibre–matrix bonding, confirming that microstructural changes directly control the macroscopic behaviour of the mortar.

### Innovative Contributions

This study combines mechanical testing and SEM analysis to assess mortars containing high levels of recycled plaster waste (up to 45%), identifying key microstructural features such as dry fronts, weak ITZs, and crack paths that govern mechanical performance. The role of polypropylene fibres in bridging cracks and improving cohesion is clearly demonstrated, including the transition from fibre pull-out to fibre rupture with matrix maturation.

## Figures and Tables

**Table 1:** State-of-the-art literature studies.

Article Title	Waste Used to Replace Aggregates/Cement	Short Summary of the Analyzed Article	Conclusions/Observations
Recycled Aggregates Influence on the Mechanical Properties of Cement-Lime-Based Mortars—Saitis Catalin et al. [8]	Old plastering mortar waste	- Cement–lime mortars with 10–15% aggregate replacement by plaster waste; - Cement-lime mortars: reference mix, 45% aggregate replacement with plaster waste, and 10% cement replacement with the same waste; - tests: bulk density, compressive & flexural strength, bond strength; - SEM analyses 28 days;	- 45% aggregate replacement causes significant strength loss (36–38% compressive; 24–30% flexural); - 10% cement replacement yields smaller strength reductions (25–30% compressive; flexural less affected); - SEM: waste particles contain hydration products but are not uniformly integrated; increased porosity and weak aggregate–matrix interfaces explain mechanical losses;
Recycling Plaster Waste as a Substitute for Aggregates in Obtaining Plastering Mortars—Saitis Catalin et al. [9]	Old plastering mortar waste	- Cement–lime mortars with 10–15% aggregate replacement by plaster waste; - fresh and hardened properties tested;	- All results obtained for the samples with 10% and 15% replacement of natural aggregates by recycled plastering mortars remain within the limits of current standards, despite showing up to a 22% reduction in mechanical strength;
Potential Use of Rendering Mortar Waste Powder as a Cement Replacement Material—A. A. Abadel et al. [10]	Rendering mortar waste powder	- Rendering mortar waste powder as partial cement replacement (0–30%); flow, strength, density, absorption, SEM	- 10–15% replacement yields moderate (10–20%) strength losses but denser microstructure; - SEM: fine powder fills pores and promotes secondary hydration;
Evaluation of Physical-Mechanical Properties of Cement–Lime Masonry Mortars Produced with Mixed Recycled Aggregates—R. L. S. Ferreira et al. [11]	Mixed recycled aggregates (brick & concrete)	- Cement–lime mortars with natural sand replaced by mixed recycled aggregates (brick + concrete);	- Moderate replacement retains strength; - SEM: porous recycled aggregates cause weaker interfacial bonding;
Properties of Cement–Lime Mortars vs. Cement Mortars Containing Recycled Concrete Aggregates—R. R. Samiei et al. [12]	Recycled concrete aggregates	- Comparison between cement–lime and cement mortars using recycled concrete aggregates	- Recycled aggregates reduce strength but can be optimized by pre-treatment; - SEM: microcracking and weak aggregate–paste bonds;
Long-Term Effect of Recycled Aggregate on Microstructure, Durability and Mechanical Behaviour of Rendering Mortars (Cement–Lime Based)—A. S. Ruviaro et al. [13]	Recycled aggregates (construction & demolition waste)	- Long-term study on recycled aggregates in cement–lime mortars.	- Capillary porosity increases with time at high waste ratios; - SEM/MIP confirm higher pore connectivity and durability concerns;
Mechanical, Durability and Sustainability Assessment of Rendering Mortar with Recycled Concrete and Ceramic Fine Aggregates—N. Garg & S. Shrivastava [14]	Recycled concrete & ceramic waste	- Cement–lime rendering mortars using concrete and ceramic fine aggregates; - mechanical and SEM tests;	- Balanced mix ratios achieve near-reference strengths; - SEM: improved aggregate bonding and reduced cracks;
High-Ductility Fiber-Reinforced Lime Mortar (NHL) with Polypropylene Fibres—R. Illampas et al. [15]	N.A.	- Natural hydraulic lime mortar reinforced with polypropylene fibers; - mechanical and SEM analyses;	- Enhanced ductility and crack control at optimal fiber dosage; - SEM: good fiber–matrix adhesion, minimal voids;
Effect of a Polypropylene Fibre on the Behaviour of Aerial Lime-Based Mortars—A. Izaguirrea [16]	N.A.	- Air-lime mortars with 0.1–0.5% polypropylene fibers; - permeability and cracking tests, SEM	- Fibers reduce cracking and permeability; - SEM: adequate bonding though poor dispersion may create voids;
Experimental Characterisation of Lime-Based Textile Reinforced Mortars (TRM)—M. Pepe et al. [17]	N.A.	- Lime-based TRM for masonry strengthening; - SEM studied fiber–matrix interface;	- Proper fiber (glass or polymer) embedding improves tensile performance; - SEM: pozzolanic additives densify matrix;
Phase Development of Lime-Based Plasters Blended with Waste Ceramic and Industrial By-Products—D. Koňáková et al. [18]	Ceramic waste and industrial by-products	- Lime plasters blended with ceramic waste and by-products; - XRD, SEM, and mechanical tests;	- Optimal replacement improves strength; - SEM/EDS: denser microstructure and new hydrate formation;
Study on Mechanical Properties and Durability of Natural Hydraulic Lime (NHL) Modified Mortars—S. Zhang et al. [19]	Pozzolanic additives (waste silica sources)	- Modified NHL mortars with pozzolanic additives; - SEM for microstructural evaluation;	- Densified matrix and increased early strength; - SEM confirms reduced porosity;
Valorization of Concrete and Brick Waste as Sustainable Mortar Components—I. Raini et al. [20]	Brick & concrete waste	- Cement–lime mortars with brick powder and concrete waste; - mechanical and SEM/MIP tests.	- Acceptable performance at moderate replacement; - SEM: porous recycled particles weaken ITZ;
Exploitation of Waste Perlite Products in Lime-Based Mortars—M. Stefanidou et al. [21]	Perlite waste	- Perlite waste in lime mortars; - workability, mechanical, and SEM tests;	- Reduces density, improves thermal insulation; - SEM: heterogeneous microstructure and open porosity;
Towards Sustainable Masonry Construction: Fine Recycled Aggregate Replacement in Cement–Lime Mortars—V. Grigorjev et al. [22]	Fine recycled aggregates (construction waste)	- Fine recycled aggregates replacing natural sand; - SEM and durability tests;	- Moderate replacement maintains strength; - SEM: porosity and bonding depend on aggregate grading;
Study of the properties of lime and cement mortars made with ceramic recycled aggregates—A. Barrios et al. [23]	Ceramic recycled aggregates	- Ceramic recycled aggregates replacing natural sand at 0–25–50% in lime-cement mortars; - tests: strength, workability, SEM.	- Up to 25% replacement retains strength; - SEM shows good integration of ceramic fragments; - higher ratios increase porosity and reduce strength;
Investigating use of dimensional limestone slurry waste as fine aggregate in mortar—P. Chouhan et al. [24]	Limestone slurry waste	- Fine aggregate replaced with limestone slurry waste up to 40% in mortar; - tests: compressive & flexural strength, SEM/MIP.	- Up to 40% replacement improved strength and maintained rheological properties; - SEM/MIP confirm denser matrix and fewer micro-cracks;
Use of Recycled Aggregates in Lime Mortars for Historical Buildings—M. Kilumile [25]	Recycled concrete & burnt-brick sand	- Fine recycled concrete sand and recycled burnt-brick sand in hydraulic lime mortars; - replacements: up to 100% sand replacement in some mixes, ages 7, 28, 45 days;	- concrete & burnt-brick sand; - Burnt-brick sand mixture yielded flexural strength +131% (7 d) and +177% (45 d) vs. control; - SEM shows pozzolanic reaction of brick fines densifying matrix;
Shrinkage and Durability of Cement Mortars with Recycled Aggregate—W. Yang et al. [26]	Recycled concrete aggregate	- Recycled aggregate (RA) replacing natural aggregate up to 50% in cement mortars; - tests: shrinkage, durability, SEM;	- Up to 50% RA replacement: shrinkage increased, but durability improved in some mixes; - SEM shows increased micro-cracking at higher RA levels;
Second Life for Recycled Concrete and Other Construction Waste in Mortars—V. Grigorjev et al. [27]	Recycled concrete & mixed CDW sand	- Life for Recycled Concrete and Other Construction Waste in Mortars; - PMC; - Review and experimental mixes replacing fine natural sand with recycled sand from concrete & mixed waste; - replacement up to 50%.	- Moderate replacement (20–30%) yields similar or improved strength; - SEM reveals improved bonding and filler effect from fine recycled sand.

**Table 2:** Materials used for preparing 1 m^3^ of each analyzed recipe.

Type of Recipe	Cement [kg/m^3^]	Aggregates [kg/m^3^]	Lime [kg/m^3^]	Water [L/m^3^]	Plaster Waste [kg/m^3^]	W/b Proportion	Polypropylene Fibers [kg]
CSIII R.S.	275	1450	110	328	0	0.85	-
CSIII 10% cement	247.5	1450	110	332	27.5	0.93	-
CSIII 45% aggregates	275	797.5	110	381	652.5	0.99	-
CSIII 45% aggregates + pp fibers	275	797.5	110	381	652.5	0.99	1.5

**Table 3:** Samples chemical composition.

Minerals/Chemical Formula	Recipe, Amount, wt. [%]			
	CS III R.S	Recycled Mortar	CS III 10% Cement	CS III 45% Aggregates
Quartz/SiO_2_	49	36	56	43
Calcite/CaCO_3_	10	25	13	18
Portlandite/Ca(OH)_2_	19	18	22	16
CSH/CaH_2_O_4_Si	22	13	9	17
Muscovite/KAl_2_(AlSi_3_O_10_) (F, OH)_2_	-	8	-	6

**Table 4:** Macroscopic and microscopic investigations.

Analyzed Characteristic	State of the Mortars	Testing Interval [Days]
Apparent density	Fresh	Immediately after preparation
	Hardened	7, 14, 28
Consistency	Fresh	Immediately after preparation
Compressive strength	Hardened	7, 14, 28
Flexural strength		
Adhesion to the support layer		28
Scanning Electron Microscopy (SEM)	Hardened	7, 14, 28

**Table 5:** Fresh state results (±Standard Deviation).

Type of Recipe	Apparent Density (Average Value) ρ_a_ [kg/m^3^] (±SD)	Consistency (Average Value) d_med_ [mm] (±SD)
CSIII R.S.	2132 ± 15 ^a^	193.5 ± 3.4 ^a^
CSIII 10% cement	2072 ± 22 ^a^	196.5 ± 1.8 ^a^
CSIII 45% aggregates	1929 ± 14 ^a^	194 ± 2.7 ^a^
CSIII 45% aggregates + pp fibers	1954 ± 12 ^a^	166.5 ± 2.8 ^b^
*p* values (Anova one way)	0.14	0.00136

The Tukey test highlights pairwise comparisons between specimens; different superscript letters (e.g., a, b) indicate statistically significant differences between groups, whereas the same subscript letter indicates that there are no statistically significant differences between groups.

**Table 6:** Hardened state results.

Type of Recipe	Testing Intervals [Days]	Apparent Density ρ_a_ [kg/m^3^]	Bending Strength f_ctm, fl_ [N/mm^2^]	Compressive Strength f_cm_ [N/mm^2^]	Adhesion to the Substrate f_u_ [N/mm^2^]
Average Values (±SD)					
CSIII R.S.	7	2121 ± 15	3.41 ± 0.6 ^a^	12.69 ± 2.1 ^a^	N.A
	14	2098 ± 14	3.66 ± 0.9 ^a^	16.04 ± 1.8 ^a^	N.A
	28	2049 ± 10	4.34 ± 1.1 ^a^	21.60 ± 2.4 ^b^	0.457 ^a^
*p*-values within the group			0.57349	≤0.05	
CSIII 10% cement	7	2051 ± 12	1.68 ± 0.2 ^a,b^	9.27 ± 0.7 ^a^	N.A
	14	2039 ± 14	2.98 ± 0.4 ^b,c^	13.15 ± 1.1 ^b^	N.A
	28	1947 ± 11	3.57 ± 0.6 ^c^	15.30 ± 1.2 ^b^	0.420 ^a^
*p*-values within the group			≤0.05	≤0.05	
CSIII 45% aggregates	7	2038 ± 13	2.12 ± 0.5 ^a,b^	9.64 ± 0.8 ^a^	N.A
	14	1918 ± 12	2.21 ± 0.3 ^b,c^	11.39 ± 1.4 ^b^	N.A
	28	1847 ± 10	3.31 ± 0.5 ^c^	13.88 ± 1.8 ^b^	0.335 ^b^
*p*-values within the group			≤0.05	≤0.05	
CSIII 45% aggregates + pp fibers	7	1943 ± 10	2.20 ± 0.3 ^a^	7.99 ± 0.6 ^a^	N.A
	14	1890 ± 11	2.56 ± 0.4 ^a^	10.27 ± 0.8 ^b^	N.A
	28	1872 ± 13	3.69 ± 0.6 ^c^	15.81 ± 0.7 ^c^	0.376 ^b^
*p*-values within the group			≤0.05	≤0.05	
*p* values between the investigated groups			0.24053	0.28482	≤0.05

The Tukey test highlights pairwise comparisons between specimens; different superscript letters (e.g., a, b, c) indicate statistically significant differences between groups, whereas the same subscript letter indicates that there are no statistically significant differences between groups.

## Data Availability

The original contributions presented in the study are included in the article.
